# Glasgow-Blatchford Score Predicts Post-Discharge Gastrointestinal Bleeding in Hospitalized Patients with Heart Failure

**DOI:** 10.3390/jcm9124083

**Published:** 2020-12-17

**Authors:** Yu Hotsuki, Yu Sato, Akiomi Yoshihisa, Koichiro Watanabe, Yusuke Kimishima, Takatoyo Kiko, Tetsuro Yokokawa, Tomofumi Misaka, Takamasa Sato, Takashi Kaneshiro, Masayoshi Oikawa, Atsushi Kobayashi, Takayoshi Yamaki, Hiroyuki Kunii, Kazuhiko Nakazato, Yasuchika Takeishi

**Affiliations:** 1 Department of Cardiovascular Medicine, Fukushima Medical University, Fukushima 960-1247, Japan; yhotsuki@fmu.ac.jp (Y.H.); yu-sato@fmu.ac.jp (Y.S.); koichi-w@fmu.ac.jp (K.W.); kimishi@fmu.ac.jp (Y.K.); tkiko@fmu.ac.jp (T.K.); yokotetu@fmu.ac.jp (T.Y.); misaka83@fmu.ac.jp (T.M.); takamasa@fmu.ac.jp (T.S.); tk2435@fmu.ac.jp (T.K.); moikawa@fmu.ac.jp (M.O.); koba-a@fmu.ac.jp (A.K.); ta-yama@fmu.ac.jp (T.Y.); hkunii@fmu.ac.jp (H.K.); nakazato@fmu.ac.jp (K.N.); takeishi@fmu.ac.jp (Y.T.); 2 Department of Advanced Cardiac Therapeutics, Fukushima Medical University, Fukushima 960-1247, Japan

**Keywords:** Glasgow-Blatchford Score, gastrointestinal bleeding, management, prognosis, heart failure

## Abstract

Background: The Glasgow-Blatchford Score (GBS) is one of the most widely used scoring systems for predicting clinical outcomes for gastrointestinal bleeding (GIB). However, the clinical significance of the GBS in predicting GIB in patients with heart failure (HF) remains unclear. Methods and Results: We conducted a prospective observational study in which we collected the clinical data of a total of 2236 patients (1130 men, median 70 years old) who were admitted to Fukushima Medical University Hospital for acute decompensated HF. During the post-discharge follow-up period of a median of 1235 days, seventy-eight (3.5%) patients experienced GIB. The GBS was calculated based on blood urea nitrogen, hemoglobin, systolic blood pressure, heart rate, and history of hepatic disease. The survival classification and regression tree analysis revealed that the accurate cut-off point of the GBS in predicting post-discharge GIB was six points. The patients were divided into two groups: the high GBS group (GBS > 6, *n* = 702, 31.4%) and the low GBS group (GBS ≤ 6, *n* = 1534, 68.6%). The Kaplan–Meier analysis showed that GIB rates were higher in the high GBS group than in the low GBS group. Multivariate Cox proportional hazards analysis adjusted for age, malignant tumor, and albumin indicated that a high GBS was an independent predictor of GIB (hazards ratio 2.258, 95% confidence interval 1.326–3.845, *p* = 0.003). Conclusions: A high GBS is an independent predictor and useful risk stratification score of post-discharge GIB in patients with HF.

## 1. Introduction

Heart failure (HF) is a systemic disease and affects other organ systems. HF causes low cardiac output, increased central venous pressure, and increased sympathetic vasoconstriction, resulting in altered gastrointestinal function [1,2,3]. Reduced intestinal perfusion may lead to an increase in transmucosal carbon dioxide pressure, and intramucosal acidosis occurs in nearly 50% of patients with circulatory failure, suggesting the presence of an inadequate oxygen supply and gastrointestinal ischemia [2,4,5]. Additionally, HF patients receive anticoagulant therapy and/or antiplatelet therapy for the management of several comorbidities such as coronary artery disease, atrial fibrillation (AF), cardiomyopathy, and valvular disease [4], leading to a high risk of bleeding [5,6]. Thus, gastrointestinal bleeding (GIB) occurs relatively easy in patients with HF [2,4,7]. In turn, GIB causes anemia, which can also cause or exacerbate HF [8,9,10] and may be associated with later higher cardiac event rates and all-cause mortality [4]. Thus, it is necessary to predict the risk of GIB, and preventing GIB is important for improving prognosis in HF patients [3]. However, risk assessment of GIB in HF patients has never been reported.

The Glasgow-Blatchford Score (GBS) is one of the most widely used scoring systems for assessment of patients at risk of GIB and was originally validated to estimate the need for emergent endoscopic intervention [11,12,13]. The GBS was originally used to assess the need for acute management in patients with upper gastrointestinal bleeding. It has also been reported that the GBS predicts the need for intervention and the high mortality rate in patients with lower GIB [14]. The GBS consists of vital signs (systolic blood pressure and heart rate), laboratory data (blood urea nitrogen and hemoglobin) and medical history (hepatic disease and HF), and these components seem to be associated with lower GIB, as well as upper GIB. On the other hand, we previously reported the prognostic impacts of GIB, which include not only upper GIB, but also lower GIB, in HF patients [4]. In the present study, we aim to clarify the predictive value of the GBS for GIB in HF patients.

## 2. Experimental Section

### 2.1. Subjects and Study Protocol

We conducted a prospective observational study of 2715 consecutive decompensated HF patients who were both admitted to and discharged from Fukushima Medical University Hospital between January 2010 and April 2019. The diagnosis of decompensated HF was defined by attending cardiologists based on the Framingham criteria and established HF guidelines [15,16,17], characterized by typical symptoms (e.g., breathlessness and fatigue) and accompanying signs (e.g., elevated jugular venous pressure, pulmonary crackles, and peripheral edema). Cardiologists decided the need for hospitalization due to decompensated and/or worsening heart failure in all cases, when management was necessary such as intravenous agents, respiratory care, dialysis, mechanical support, etc. We excluded 180 patients who were receiving maintenance dialysis and 299 patients who lacked data on the history of liver disease. It has been reported that gastrointestinal bleeding is frequent regardless of the GBS in dialysis patients, [18,19], and these patients were excluded in the present study. GIB was defined as bleeding from the gastrointestinal tract with Type 2 to 5 bleeding according to the Bleeding Academic Research Consortium (BARC) definition [4,20]. The sources of GIB were detected by endoscopy, imaging techniques such as computed tomography, and surgery, as much as possible. Only post-discharge bleeding was counted. The GBS consists of vital signs (systolic blood pressure and heart rate), laboratory data (blood urea nitrogen and hemoglobin), and medical history (hepatic disease and HF), and each item is assigned 0 to 6 points (in total, 0 to 23 points) [11,21]. These assessments were performed in a stable condition prior to hospital discharge by study team members. In HF patients, vital signs and laboratory data drastically change during hospitalization. In the present study, we wanted to examine the predictive value of the GBS prior to discharge on GIB during the post-discharge follow-up period. The score was calculated for each patient and determined by the total points. In this study, all patients had HF, and all were given at least 2 points (in total, 2 to 17 points). Next, the best cut-off points of the GBS for GIB were explored using survival classification and regression tree (CART) analysis [22]. This analysis uses predictor variables (the GBS), outcome variables (GIB), and period until the event occurs, and the analysis revealed the accurate cut-off value of the GBS in predicting post-discharge GIB. Based on the cut-off points, we divided the residual 2236 patients into two groups: the high and the low GBS group.

The patient characteristics included age, sex, body mass index, comorbidity, medications, laboratory data, and echocardiographic data. Comorbidity was defined as previously reported [23,24]. These assessments were performed during hospitalization. The patients were followed up until March 2020 for the occurrence of GIB. Since these patients visited their referring hospital monthly or bi-monthly, we were able to follow up on all patients. The status and dates of GIB of all patients were obtained from the patients’ medical records, attending physicians at the patient’s referring hospitals, or by contacting the patients by telephone. Written informed consent was obtained from all patients. The study protocol was approved by the ethical committee of Fukushima Medical University. The investigation conforms with the principles outlined in the Declaration of Helsinki, and reporting of the study conforms to Strengthening the Reporting of Observational Studies in Epidemiology guideline [25].

### 2.2. Statistical Analysis

The Shapiro–Wilk test showed that all continuous variables were non-parametric and were presented as a median (interquartile range). Categorical variables were expressed as numbers and percentages. Continuous variables were compared using the Mann–Whitney U test, and the chi-squared test was used for comparisons of categorical variables. The Kaplan–Meier analysis was used for presenting post-discharge GIB, and the log-rank test was used for initial comparisons. The adjusted hazards ratios (HRs) and 95% confidence intervals (CIs) of each variable associated with GIB were calculated by the Cox proportional hazards model. In the univariate Cox proportional hazards analysis, we adjusted for the following possible confounding factors that differed between the high and low GBS groups: age, sex, body mass index, diabetes mellitus, malignancy, B-type natriuretic peptide, creatinine, albumin, and platelet. Because of the multicollinearity between the GBS and its components (systolic blood pressure, heart rate, blood urea nitrogen, hemoglobin, and hepatic disease), the components were not included in the Cox proportional hazard analysis. A *p*-value of <0.05 was considered statistically significant for all comparisons. These analyses were performed using statistical software packages (SPSS ver. 24.0, IBM, Armonk, NY, USA; EZR ver. 1.37, Saitama Medical Center, Jichi Medical University, Saitama, Japan) [26]. EZR is for R. More precisely, it is a modified version of the R commander designed to add statistical functions frequently used in biostatistics.

## 3. Results

In the follow-up period (mean 1235 days), seventy-eight patients (3.5%) developed GIB. The BARC classification of these patients was Type 2 (*n* = 16, 20.5%), Type 3 (*n* = 44, 56.4%), Type 4 (*n* = 3, 3.8%), and Type 5 (*n* = 7, 9.0%). The sources of GIB were determined by endoscopy (*n* = 45, 57.7%), computed tomography (*n* = 7, 8.9%), and surgery (*n* = 18, 23.1%) and remained unknown in eight patients (10.2%). The sources of GIB were in the esophagus (esophagitis, varix, Mallory–Weiss syndrome; *n* = 6, 7.6%), stomach (cancer, ulcer, polyp, etc.; *n* = 17, 21%), duodenum (ulcer; *n* = 4, 5.1%), small intestine (telangiectasia; *n* = 5, 6.4%), colon (cancer, ulcer, polyp, diverticulum, ischemic colitis, etc.; *n* = 29, 37.1%), and unknown source (*n* = 3, 3.9%). The survival CART analysis revealed that the accurate cut-off value of the GBS in predicting post-discharge GIB was six (follow-up period, mean 1420 days, median 1235 days, range 1–3704 days). In addition, the GBS with a cut-off value of six predicted upper GIB (area under the curve 0.68, sensitivity 0.69, specificity 0.64) and lower GIB (area under the curve 0.72, sensitivity 0.75, specificity 0.68). Thus, the GBS was useful for predicting for lower GIB, as well as upper GIB in the present study. We divided the 2236 patients into two groups: the high GBS group (GBS > 6, *n* = 702, 31.4%) and the low GBS group (GBS ≤ 6, *n* = 1534, 68.6%). Comparisons of the clinical characteristics are shown in Table 1. Compared with the low GBS group, the high GBS group had higher age and heart rate and lower body mass index and systolic blood pressure. The high GBS group had a higher prevalence of comorbidities such as diabetes mellitus, malignant disease, and hepatic disease. Medications including antiplatelet agents, anticoagulants, and non-steroidal anti-inflammatory drugs did not significantly differ between the two groups. The levels of B-type natriuretic peptide, creatinine, and blood urea nitrogen were higher, while hemoglobin, albumin, and platelet were lower in the high GBS group than in the low GBS group. In contrast, the left ventricular ejection fraction had no significant differences between the two groups.

**Figure 1:**
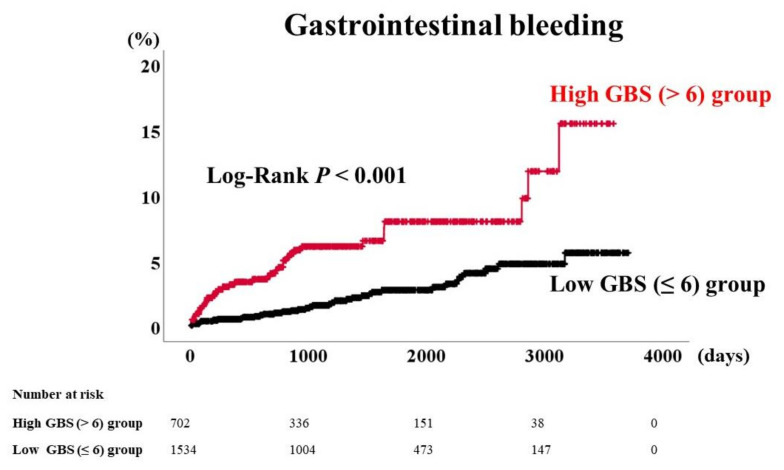
Kaplan–Meier analysis for gastrointestinal bleeding rates stratified by the GBS levels. GBS indicates Glasgow-Blatchford Score.

In the Kaplan–Meier analysis (Figure 1), the occurrence of GIB was significantly higher in the high GBS group (log-rank *p* < 0.001). Next, we performed the univariate Cox proportional hazards analysis (Table 2). We considered possible confounding factors as follows: age, sex, body mass index, diabetes mellitus, malignant disease, B-type natriuretic peptide, creatinine, albumin, and platelet, which differed between the low GBS and high GBS groups. In the analysis, age, malignant disease, albumin, and the GBS were associated with post-discharge GIB (Table 2). Furthermore, multivariate Cox proportional hazards analysis after adjusting for the above confounding factors showed that a high GBS (hazards ratio 2.258, 95% confidence interval 1.326–3.845, *p* = 0.003) and the presence of malignant disease were independent predictors of GIB.

## 4. Discussion

To the best of our knowledge, the present study is the first to report that a high GBS is an independent predictor and a useful risk stratification scoring system of post-discharge GIB in patients with HF. The survival CART analysis determined that the optimal cut-off point was six points.

There are several risk-scoring systems to assess patients presenting with GIB and to predict the need for treatment for GIB (e.g., emergent endoscopic intervention) [11,12,27,28,29,30]. Higher GBSs were associated with higher rebleeding rates following discharge in hospitalized patients with upper GIB [13,31]. Some scoring systems require performing endoscopy. The Rockall score is commonly used, but requires endoscopic findings (e.g., signs of bleeding, Mallory–Weiss syndrome, and malignancy) [12,27]. On the contrary, the GBS is based on simple clinical and laboratory parameters, does not require performing endoscopy, and is useful to non-gastroenterologists (i.e., cardiologists) [12]. In addition, the GBS has been shown to be superior to the Rockall score in the assessment of in-hospital rebleeding and mortality, as well as the need for emergency endoscopic interventions and blood transfusions [11,12,32,33,34].

The GBS was originally used to identify low risk patients with a GBS score of zero who are unlikely to need endoscopic intervention [11,32]. Banister et al. reported that patients with acute upper GIB and a low GBS (namely, GBS of ≤1) can be discharged from the emergency department [21]. However, the score is no longer useful in predicting which patients require therapeutic endoscopy among those who do not have a low GBS [35]. Influenced by that, Bryant et al. performed a prospective study over 24 months on consecutive hospitalized patients with upper GIB [35]. Patients with a higher GBS had a higher rate of endoscopic intervention, blood transfusion, rebleeding, repeat endoscopy, or mortality, compared to those with a lower GBS. Based on the Receiver Operating Characteristic (ROC) analysis, the optimal cut-off value of the GBS was seven, which increased the risk of significant bleeding requiring endoscopic and surgical intervention, as well as the risk of death [35]. Sengupta et al. conducted a prospective observational cohort study of patients who were admitted with upper GIB or had bleeding during hospitalization and were followed up for 30 days after discharge. They reported that patients with a high GBS (>7) at admission had higher rebleeding rates following discharge [13]. In the present study, the optimal cut-off was six points, which was relatively similar to the above reports [13,35].

In the present study, patients with a high GBS were at increased risk of GIB after discharge even in HF patients, with a longer follow-up period (median 1235 days) than previous reports [13,18,31]. Previous studies have shown that the GBS is expected to be used in the emergency department or post-hemostasis where the vital signs are unstable during hospitalization or the early phase after discharge [13,21], whereas the present study showed that the GBS may be useful in the chronic phase of HF after discharge. Furthermore, many previous reports targeted acute GIB patients, and the endpoint was almost rebleeding [13,34]. On the contrary, the present study evaluated post-discharge GIB in patients with HF, including patients with the first occurrence of GIB. Thus, the GBS may be effective at predicting GIB in patients with HF, and HF patients with a high GBS (>6) should be carefully managed to prevent the occurrence of GIB. In HF patients with a high GBS, it would be necessary to reconsider the adequacy of the administration of antiplatelet drugs and anticoagulants and use acid suppressors as needed [36,37]. Furthermore, predicting GIB could potentially lead to the reduction of the risk of cardiac events and all-cause mortality [4].

## 5. Study Limitations

The present study has several limitations. First, as a prospective cohort study of a single center with a relatively small number of patients, the results may not be representative of the general HF population. Second, the present study included variables during hospitalization for decompensated HF, without taking into consideration changes in medical parameters (e.g., the GBS) and post-discharge treatment. Therefore, the present results should be viewed as preliminary, and further studies with a larger population are needed.

## 6. Conclusions

The GBS can be a useful predictor of GIB in patients with HF. HF patients with a high GBS should be carefully managed to prevent the occurrence of GIB.

## Figures and Tables

**Table 1:** Baseline patient characteristics (*n* = 2236).

	Low GBS Group (*n* = 1534, 68.6%)	High GBS Group (*n* = 702, 31.4%)	*p*-Value
GBS	3.5 (2.0–5.0)	9.0 (7.0–10.0)	<0.001
Demographic data			
Age (years old)	67.0 (57.0–75.0)	74.5 (65.0–81.0)	<0.001
Male sex (*n*, %)	913 (59.5)	417 (59.4)	0.959
Body mass index (kg/m^2^)	23.2 (20.8–26.0)	22.1 (19.6–24.6)	<0.001
HR at discharge (/min)	72.5 (62.0–87.0)	78.0 (65.0–92.0)	<0.001
SBP at discharge (mmHg)	123.0 (110.0–138.0)	115.0 (98.0–135.0)	<0.001
Comorbidities			
Hypertension (*n*, %)	1023 (66.7)	488 (69.5)	0.185
Diabetes mellitus (*n*, %)	528 (34.4)	331 (47.2)	<0.001
Dyslipidemia (*n*, %)	1085 (70.7)	486 (69.2)	0.472
Coronary artery disease (*n*, %)	465 (30.3)	225 (32.1)	0.409
Malignant disease (*n*, %)	260 (17.5)	188 (27.8)	<0.001
Hepatic disease (*n*, %)	172 (11.2)	166 (23.6)	<0.001
Medications at discharge			
β blockers (*n*, %)	1109 (72.3)	506 (72.1)	0.916
ACEIs/ARBs (*n*, %)	1079 (70.3)	489 (69.7)	0.744
Antiplatelet agents (*n*, %)	783 (51.0)	350 (49.9)	0.603
Anticoagulants (*n*, %)	878 (57.2)	406 (57.8)	0.790
Warfarin (*n*, %)	627 (40.9)	307 (43.7)	0.203
DOACs (*n*, %)	311 (20.3)	128 (18.2)	0.260
NSAIDs (*n*, %)	60 (4.0)	32 (4.6)	0.501
PPI	930 (60.6)	485 (69.1)	<0.001
H_2_ blocker	117 (11.5)	87 (12.4)	0.561
Laboratory data			
B-type natriuretic peptide (pg/mL)	166.5 (59.3–404.0)	421.6 (146.4–818.2)	<0.001
Creatinine (mg/dL)	0.84 (0.70–1.00)	1.20 (0.91–1.56)	<0.001
Blood urea nitrogen (mg/dL)	16.0 (13.0–20.0)	27.0 (20.0–35.0)	<0.001
Hemoglobin (g/dL)	13.6 (12.5–14.8)	10.7 (9.5–12.0)	<0.001
Albumin (g/dL)	4.0 (3.7–4.3)	3.5 (3.1–3.9)	<0.001
Platelet count (10^3^/µL)	196.0 (163.0–236.0)	168.0 (130.0–214.0)	<0.001
Echocardiographic data			
LVEF (%)	56.3 (40.0–64.4)	52.8 (38.3–63.2)	0.218

GBS, Glasgow-Blatchford Score; HR, heart rate; SBP, systolic blood pressure; ACEI, angiotensin-converting enzyme inhibitor; ARB, angiotensin receptor blocker; DOAC, direct oral anticoagulant; NSAID, non-steroidal anti-inflammatory drug; LVEF, left ventricular ejection fraction; PPI, proton pump inhibitor.

**Table 2:** Univariate Cox proportional hazards analysis for gastrointestinal bleeding (78 events).

	Univariate			Multivariate		
	HR	95% CI	*p*-Value	HR	95% CI	*p*-Value
Age (per 1 year increase)	1.030	1.011–1.049	0.002	1.016	0.995–1.037	0.131
Male sex	1.288	0.808–2.055	0.287			
BMI (per 1 kg/m^2^ increase)	0.968	0.914–1.025	0.267			
Diabetes mellitus	1.089	0.693–1.710	0.712			
Malignant disease	3.139	1.994–4.943	<0.001	2.357	1.430–3.089	0.001
Log-BNP	1.384	0.925–2.068	0.114			
Creatinine (per 1 mg/dL increase)	1.135	0.839–1.534	0.411			
Albumin (per 1 g/dL increase)	0.575	0.403–0.820	0.002	0.790	0.525–1.188	0.258
Platelet count (per 10^3^/µL increase)	0.996	0.993–1.000	0.052			
GBS over 6 points	2.868	1.836–4.478	<0.001	2.258	1.326–3.845	0.003

HR, hazard ratio; CI confidence interval; BMI, body mass index; BNP, B-type natriuretic peptide; GBS, Glasgow-Blatchford Score.
